# Sex differences in neurodevelopmental trajectories in children with different levels of autistic traits

**DOI:** 10.1111/pcn.13529

**Published:** 2023-02-06

**Authors:** Tomoko Nishimura, Nagahide Takahashi, Akemi Okumura, Taeko Harada, Toshiki Iwabuchi, Chikako Nakayasu, Mohammad Shafiur Rahman, Satoshi Uchiyama, Manabu Wakuta, Yoko Nomura, Nori Takei, Atsushi Senju, Kenji J. Tsuchiya

**Affiliations:** ^1^ Research Center for Child Mental Development Hamamatsu University School of Medicine Hamamatsu Japan; ^2^ United Graduate School of Child Development Hamamatsu University School of Medicine Hamamatsu Japan; ^3^ Department of Child and Adolescent Psychiatry Nagoya University Graduate School of Medicine Nagoya Japan; ^4^ Center for Consultation and Support for Developmental Disorders Hamamatsu Japan; ^5^ Institute of Child Developmental Science Research Hamamatsu Japan; ^6^ Queens College and Graduate Center City University of New York New York City New York USA

**Keywords:** autism spectrum disorder, child development, communication, language development, sex characteristics

## Abstract

**Aim:**

Little is known about early manifestations of autism spectrum disorders (ASD) in females, including those who may be overlooked by the current diagnostic criteria. We longitudinally explored sex differences in the trajectories of cognitive and motor functions and adaptive behaviors in children with different levels of autistic traits.

**Methods:**

The participants were 824 children from the Hamamatsu Birth Cohort for Mothers and Children (HBC Study), Japan, who were classified into three autistic trait groups—low, moderate, and high—based on the Social Responsiveness Scale–Second Edition. Cognitive and motor functions were measured at seven time‐points from 0.5 to 3.5 years of age using the Mullen Scales of Early Learning. Adaptive behaviors were measured at five time‐points from 2.7 to 9 years of age using the Vineland Adaptive Behavior Scales–Second Edition. Trajectories were depicted using latent growth curve modeling.

**Results:**

Sex‐specific trajectories were observed in the high‐autistic‐trait group, with only males showing a temporary decline in expressive language around the age of 2 years and a slight improvement thereafter. They also showed a slight improvement around 3 years in the adaptive behavior communication domain but a gradual downward trend later. Females in the high‐autistic‐trait group showed no distinct manifestation before the age of 3 years but showed a downward trend after 3.5 years in the adaptive behavior communication domain.

**Conclusion:**

Females and males with higher autistic traits than their same‐sex peers, independent of clinical diagnosis, may have different phenotypes in certain neurodevelopmental domains during infancy and early childhood.

Autism spectrum disorders (ASD) involve complex, early‐onset neurodevelopmental conditions. The effectiveness of early intervention has been demonstrated in several studies,^1^, ^2^ highlighting the need for early detection and diagnosis. The prevalence of ASD among children aged 8 years was reported to be 23.0 per 1000 (one in 44) in the United States.^3^ ASD has been consistently found to be more common in males, with a male‐to‐female ratio of approximately 4:1.^4^ Various studies have proposed that sexually dimorphic factors such as genetics, sex hormones, and immune function are involved in the sex difference in the prevalence of ASD, but the mechanisms underlying vulnerability and protection for males and females, respectively, remain unknown.^5^, ^6^

One of the major factors reported to influence the male‐to‐female ratio of ASD was co‐occurring intellectual disability (ID).^7^ The reported ratio was 6–16:1 for individuals without ID and approximately 1–2:1 for those with moderate to severe ID.^4^ These reports suggest that co‐occurring ID increases females' likelihood of receiving an ASD diagnosis; conversely, females without ID may be under‐diagnosed.^8^, ^9^ The current diagnostic criteria are primarily based on the male manifestation of ASD, and therefore assessments may not be sensitive enough to detect manifestation in females, especially those without ID.^10^, ^11^

Most of the autism rating scales developed to establish caseness in a clinical sense have been based on a qualitative measure of autistic traits (behavioral‐cognitive traits linked to autism),^12^ and those traits have often been derived from male‐dominated studies. Nevertheless, it has become increasingly recognized that some qualitative autistic traits of females differ from those of males: females show greater awareness of the need for social interaction than males, passivity often perceived as “just being shy,” and restricted interest in interaction with people and animals—these may be perceived as less associated with ASD.^10^, ^13^

Scales to quantitatively measure autistic traits have also been developed, based on the conception that autistic traits are continuously distributed across the population and that autistic disorder represents the upper extreme of a constellation of deficits.^12^ Such scales have been standardized in the general population with equal numbers of females and males. Using these scales, the distribution of autistic traits was reported to have sex differences, with the female population having a smaller mean than the male population.^14^

Most studies, however, have found minimal sex differences in quantitative and qualitative autistic traits in individuals with a clinical diagnosis of ASD.^11^, ^15^ Note that these studies only included females who were already diagnosed with ASD. Hence, females who might have been overlooked by the current diagnostic criteria were omitted from the study.

Another factor that should be considered is the later age of diagnosis, and possible underdiagnosis, for females. It is often reported that females are diagnosed with ASD at a later age than males.^16^, ^17^ Bargiela *et al*. reported that females diagnosed in late adolescence or adulthood experienced one or more mental health difficulties, with anxiety, depression, and eating disorders being the most commonly reported.^18^ Individuals with undiagnosed or late‐diagnosed ASD, regardless of sex, may miss opportunities to receive appropriate early interventions that would prevent them from developing co‐occurring psychiatric symptoms.

The current study focuses on sex differences in early signs of ASD, with the hope that this line of research could help mitigate the current problem of late diagnosis and underdiagnosis. Importantly, although it has been suggested that females with autistic traits and without ID are overlooked by the current diagnostic criteria,^11^ samples of studies examining sex differences in early signs have not involved females with autistic traits not meeting the current diagnostic criteria for ASD. Early signs of ASD should also be examined in females with autistic traits below the current diagnostic threshold.

Therefore, in the present study, we aim to longitudinally explore sex differences in early signs of autistic traits in a general population birth cohort, to address the abovementioned knowledge gap. The study includes females and males with different levels of autistic traits—low, moderate, and high. These autistic trait groups are classified based on the Social Responsiveness Scale–Second Edition (SRS–2)^19^; this scale provides a continuous measure of social ability and helps us identify individuals with subclinical autistic traits. Autistic traits were measured at 9 years of age; this age is above the average age of ASD diagnosis, including Asperger's syndrome, for both females and males.^16^ Some of the children, especially females, in the high autistic trait group would not meet the current diagnostic criteria for ASD; however, they would have quantitatively more autistic traits compared to same‐sex peers. We predicted that females in the high‐autistic‐trait group would show less early motor and cognitive decline than their male counterparts. We also predicted that females, as well as males, in this group would exhibit difficulties in adaptive behaviors, especially at school ages.

## Methods

### Study design and participants

This study was conducted as part of an ongoing prospective cohort study—the Hamamatsu Birth Cohort Study for Mothers and Children (HBC Study)—comprising mothers (*n* = 1138) and their children (*n* = 1258).^20^, ^21^ The HBC Study invited all women in the first or second trimester of pregnancy and who visited the Hospital of Hamamatsu University School of Medicine or the Kato Maternity Clinic between November 2007 and March 2011. The majority (99%) of enrolled mothers were Japanese. The participating children and mothers were invited for assessments when the children were aged 0.5, 0.8, 1.2, 1.5, 2, 2.7, 3.5, 4.5, 6, and 9 years. Participants of this study were 826 children who completed the assessment at 9 years. Two children diagnosed with Down syndrome were excluded from the analyses; finally, 824 children and 746 mothers were included. The participating children were representative of the general Japanese population regarding demographic characteristics and standardized T‐scores (i.e., mean of T‐scores and standard deviations (SDs); Table S1, in Appendix S1). Further details of the study have been previously described.^21^

All procedures contributing to this work comply with the ethical standards of the relevant national and institutional committees on human experimentation and with the Declaration of Helsinki, 1975, as revised in 2008. All procedures were approved by the Institutional Review Board of the Hamamatsu University School of Medicine (No. 18‐166, 19‐9, 20‐82, 22‐29, 24‐67, 24‐237, 25‐143, 25‐283, E14‐062, E14‐062‐1, E14‐062‐3, 17‐037, 17‐037‐3, 20‐233). Written informed consent was obtained from all mothers for their own as well as their children's participation.

### Measures

#### Autistic traits

The SRS–2 School Age Form^19^ was used to measure autistic traits at 9 years of age. The SRS–2 comprises 65 items and is completed by a parent or caregiver who has routinely observed the child in their natural social settings. Item response scores range from 0 (not true) to 3 (almost always true). The SRS–2 was developed to capture autistic traits that are continuously distributed in the general population, and its scores have been shown to approximate a normal distribution. As the present study was conducted in the general population, the SRS–2 was selected to measure autistic traits. The SRS–2 raw scores were converted to T‐scores (mean = 50, SD = 10)—normalized based on a nationally representative standardization sample stratified by sex.^14^ A T‐score ≥76 is considered as high autistic traits, indicative of the clinical diagnosis of ASD. T‐scores of 66 to 75 are interpreted as a moderate range of deficiencies that are clinically significant and lead to substantial interference in everyday social interactions. T‐scores of 60 to 65 are in the mild range, indicating mild to moderate deficits in social interaction. T‐scores ≤59 are considered within typical limits (low autistic traits) and are generally not associated with clinically significant ASD.^14^ In this study, the moderate and mild ranges were combined into one group because children with scores in these ranges were considered to have autistic traits below the clinical diagnostic threshold of ASD.

#### Motor and cognitive functions

Early motor and cognitive functions extending from early infancy to early childhood were assessed using the Mullen Scales of Early Learning (MSEL)^22^ at seven time‐points between 0.5 and 3.5 years of age. The MSEL is a composite scale used to assess motor and cognitive functions by direct testing and comprises five domains: gross motor, fine motor, visual reception, receptive language, and expressive language. The raw scores were converted into standard scores,^23^ which were age‐adjusted normative scores. Higher scores indicated better motor and cognitive progress.

#### Adaptive behaviors

Everyday functional abilities from early childhood to school ages were quantified using the Japanese version of the Vineland Adaptive Behavior Scales–Second Edition (VABS‐II)^24^, ^25^ at 2.7, 3.5, 4.5, 6, and 9 years of age. The VABS‐II is based on a semi‐structured parental interview comprising four domains: communication, daily living, socialization, and motor skills. We obtained age‐adjusted standard scores for the three domains of communication, daily living, and socialization. Higher scores indicated better adaptive behaviors.

#### Potential confounders

As potential confounders, the following variables, which could influence autistic traits and/or neurodevelopment, were selected: intelligence quotient (IQ), assessed using the Japanese version of the Wechsler Intelligence Scale for Children–Fourth Edition (WISC–IV)^26^ at 9 years; clinical diagnosis of ASD at 32 months of age; background characteristics of children including birthweight, gestational age at birth, and birth order; and background characteristics of parents including parental age at birth, educational history, and annual household income at birth. Next, we developed a directed acyclic graph (DAG)^27^ to select a minimally sufficient set of confounders based on the existing literature on causal relationships. All variables, except IQ and clinical diagnosis, were included in the following statistical analyses as confounders (Fig. S1, in Appendix S1).

### Statistical analysis

Developmental trajectories of motor and cognitive functions and adaptive behaviors were estimated separately using latent growth curve modeling (LGCM). Using LGCM, growth variations of initial status (intercept) and growth rate (slope and quadratic term) can be captured in the course of time (Fig. S2, in Appendix S1). A model with growth parameters of intercept and slope was compared to a model with a quadratic term in addition to these two parameters based on the model fit indices of posterior predictive *P*‐value (PPP) and deviance information criterion (DIC).^28^ A model with PPP <0.10 and ∆DIC >7 was evaluated to fit better.^29^ In terms of these model fits, quadratic terms were included for both neurodevelopment and adaptive behaviors. The main effects of autistic trait group and sex and the interactions of these variables were estimated using the Bayes estimator.

Missing values were found in 6.8% of the data for the MSEL and 2.6% for the VABS‐II. These percentages did not differ among autistic‐trait groups and sexes. As there were no crucial problems in the pattern of missingness, missing values were handled using multiple imputation by chained equations, assumed to be missing at random. Missing values were imputed to create 20 complete data sets. All variables including interaction terms were included in the imputation model. These data sets were created separately for each domain of the MSEL and VABS‐II; then, the results were integrated to obtain a summarized estimation. All analyses were conducted using Mplus version 8.5.^30^

## Results

Based on the T‐scores of the SRS–2, female and male participants were classified into three autistic trait groups for low (female: *n* = 345, 85.6%; male: *n* = 347, 82.4%), moderate (female: *n* = 46, 11.4%; male: *n* = 63, 15.0%), and high (female: *n* = 12, 3.0%; male: *n* = 11, 2.6%). Sample characteristics of each group are shown in Table 1. Distributions of raw scores and T‐scores of the SRS–2, are shown in Figs. S3 and S4, in Appendix S1, respectively; sex differences in each autistic trait group and group differences in demographic characteristics are shown in Tables S2 and S3, in Appendix S1, respectively.

**Table 1:** Demographic characteristics of children in each autistic‐trait group

	Female (*n* = 403)			Male (*n* = 421)		
Characteristic	Low (*n* = 345, 85.6%)	Moderate (*n* = 46, 11.4%)	High (*n* = 12, 3.0%)	Low (*n* = 347, 82.4%)	Moderate (*n* = 63, 15.0%)	High (*n* = 11, 2.6%)
SRS‐2 total raw score; mean (SD)	26.0 (10.7)	57.4 (7.0)	84.0 (12.3)	30.9 (11.5)	63.0 (8.3)	95.8 (11.3)
SRS‐2 total T‐score; mean (SD)	47.0 (6.2)	65.4 (4.0)	81.0 (7.0)	48.3 (6.0)	65.1 (4.3)	82.4 (5.8)
WISC‐IV full scale IQ; mean (SD)	104.1 (12.7)	98.5 (16.0)	91.2 (11.2)	101.0 (13.9)	98.3 (15.8)	91.7 (16.4)
Background characteristics of children						
Birthweight (g); mean (SD)	2872.5 (439.3)	2932.1 (444.0)	2897.5 (370.5)	2966.8 (471.3)	2972.7 (503.7)	3031.5 (505.8)
Gestational age (week); mean (SD)	38.9 (1.6)	39.1 (1.5)	38.8 (1.4)	38.8 (1.7)	39.0 (1.7)	39.0 (1.1)
Parity: primipara; *n* (%)	173 (50.1)	28 (60.9)	8 (66.7)	160 (46.1)	37 (58.7)	7 (63.6)
Parity: multipara; *n* (%)	172 (49.9)	18 (39.1)	4 (33.3)	187 (53.9)	26 (41.3)	4 (36.4)
Background characteristics of parents						
Mother's age at birth (y); mean (SD)	31.8 (4.9)	33.1 (5.4)	31.4 (6.2)	31.9 (5.0)	32.4 (5.6)	31.3 (4.0)
Father's age at birth (y); mean (SD)	33.7 (5.7)	35.3 (5.9)	36.9 (5.2)	33.2 (5.6)	34.7 (6.8)	32.9 (5.6)
Mother's years of education (y); mean (SD)	14.1 (1.9)	13.7 (1.9)	13.3 (1.7)	14.0 (1.9)	13.8 (1.5)	13.8 (1.3)
Father's years of education (y); mean (SD)	14.3 (2.6)	14.0 (2.7)	12.7 (2.7)	14.1 (2.5)	14.0 (3.1)	14.3 (2.0)
Annual household income (million JPY); mean (SD)	6.5 (0.3)	6.3 (0.3)	5.6 (0.2)	5.9 (0.3)	5.9 (0.3)	6.0 (0.2)

Abbreviations: SRS‐2, Social Responsiveness Scale, Second Edition; WISC‐IV, Wechsler Intelligence Scale for Children‐Fourth Edition.

### Trajectories of motor and cognitive functions

**Fig. 1:**
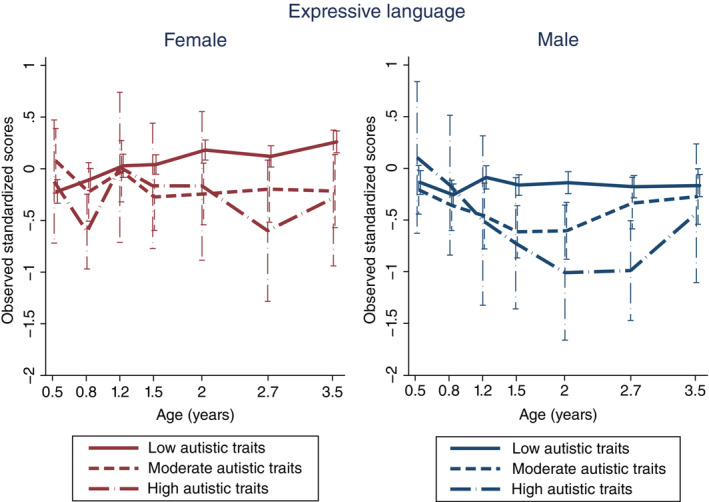
Observed trajectories of expressive language in females and males in each autistic‐trait group.

In LGCM, the group‐by‐sex interactions were significant in expressive language in the high‐autistic‐trait group (slope: *β* = 1.04, 95% confidence interval [CI]: 0.06, 2.31; quadratic: *β* = −0.33, 95% CI: −0.70, −0.01; Table 2). For males in this group, the decline in expressive language scores grew wider up to approximately 2 years, but with age the scores got closer to those in the low‐autistic‐trait group (observed standardized scores and SDs are shown in Fig. 1). However, females in the high‐autistic‐trait group did not demonstrate an apparent decline in expressive language. In expressive language, the main effects of autistic‐trait groups were significant; both moderate and high groups had negative slopes and positive quadratic terms (suggesting U‐shaped trajectories) compared with the low‐autistic‐trait group (Table 2). The main effect of female sex was positive for the slope in expressive language (suggesting upward trajectories). The interactions of group and sex were not significant in any other domains of the MSEL. Detailed results of LGCM on cognitive and motor functions are shown in Table 2, and estimated and observed trajectories are shown in Figs. S5 and S6, in Appendix S1, respectively.

**Table 2:** Estimated standardized coefficients in each domain of MSEL using the latent growth curve modeling

*β* (95% CI)					
	Gross motor	Fine motor	Visual reception	Receptive language	Expressive language
Female sex by moderate‐autistic‐trait group interactions					
Intercept	0.15 (−0.25, 0.49)	0.01 (−0.31, 0.32)	0.13 (−0.23, 0.43)	0.16 (−0.19, 0.46)	0.22 (−0.13, 0.60)
Slope	−0.36 (−0.91, 0.21)	−0.04 (−0.58, 0.50)	−0.26 (−0.76, 0.33)	−0.14 (−0.60, 0.37)	0.07 (−0.47, 0.62)
Quadratic	0.09 (−0.08, 0.27)	0.01 (−0.16, 0.17)	0.09 (−0.11, 0.25)	0.04 (−0.12, 0.17)	−0.07 (−0.23, 0.08)
Female sex by high‐autistic‐trait group interactions					
Intercept	−0.24 (−1.07, 0.53)	−0.06 (−0.75, 0.64)	0.09 (−0.58, 0.77)	0.09 (−0.78, 0.78)	−0.36 (−1.15, 0.27)
Slope	0.75 (−0.53, 1.98)	−0.32 (−1.47, 0.85)	0.004 (−1.26, 1.16)	0.82 (−0.17, 2.04)	1.04 (0.06, 2.31)*
Quadratic	−0.13 (−0.51, 0.24)	0.16 (−0.22, 0.50)	0.02 (−0.38, 0.40)	−0.33 (−0.68, −0.03)	−0.33 (−0.70, −0.01)*
Main effects of moderate‐autistic‐trait group (low is reference)					
Intercept	−0.11 (−0.36, 0.14)	−0.10 (−0.33, 0.11)	−0.06 (−0.25, 0.16)	−0.02 (−0.25, 0.23)	−0.09 (−0.33, 0.14)
Slope	0.15 (−0.29, 0.49)	0.03 (−0.32, 0.39)	−0.20 (−0.55, 0.14)	−0.26 (−0.57, 0.07)	−0.45 (−0.84, −0.08) *
Quadratic	−0.06 (−0.17, 0.08)	−0.03 (−0.14, 0.09)	0.06 (−0.05, 0.18)	0.08 (−0.01, 0.18)	0.16 (0.05, 0.28) *
Main effects of high‐autistic‐trait group (low is reference)					
Intercept	0.19, (−0.40, 0.84)	−0.04 (−0.60, 0.51)	−0.25 (−0.74, 0.25)	−0.20 (−0.69, 0.45)	0.34 (−0.16, 0.93)
Slope	−0.97 (−1.86, −0.02)*	−0.05 (−0.90, 0.89)	0.17 (−0.67, 1.06)	−0.61 (−1.49, 0.14)	−1.22 (−2.21, −0.51)*
Quadratic	0.20 (−0.06, 0.48)	−0.04 (−0.33, 0.22)	−0.06 (−0.34, 0.23)	0.25 (0.02, 0.49)*	0.37 (0.16, 0.66)*
Main effects of female sex (male is reference)					
Intercept	−0.07 (−0.19, 0.08)	0.02 (−0.11, 0.16)	−0.08 (−0.21, 0.04)	−0.04 (−0.18, 0.08)	−0.01 (−0.14, 0.11)
Slope	0.03 (−0.19, 0.24)	0.18 (−0.05, 0.39)	0.26 (0.04, 0.45)*	0.38 (0.20, 0.61)*	0.26 (0.06, 0.44)*
Quadratic	0.03 (−0.04, 0.10)	0.002 (−0.07, 0.07)	−0.05 (−0.11, 0.01)	−0.10 (−0.17, −0.04)*	−0.05 (−0.11, 0.01)

Abbreviations: β, standardized coefficient; MSEL, Mullen Scales of Early Learning; 95% CI, 95% confidence interval.

* *p* < 0.05.

### Trajectories of adaptive behaviors

**Fig. 2:**
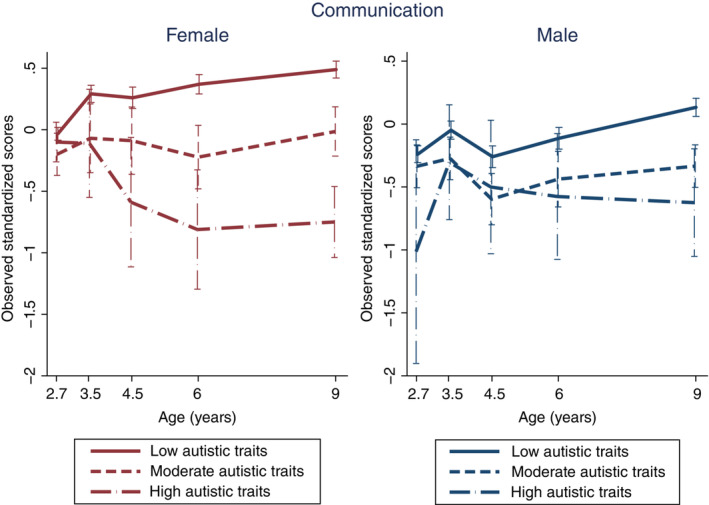
Observed trajectories of communication in females and males in each autistic‐trait group.

In the communication domain of adaptive behavior, the group‐by‐sex interactions were significant for the intercept, slope, and quadratic terms in the high‐autistic‐trait group (Table 3). In this group, females showed a higher initial score than their male counterparts and a downward trend after 3.5 years of age (observed standardized scores and SDs are shown in Fig. 2). Males in this group showed a slight improvement up to the age of 3.5 years, but a gradual decline thereafter. The main effects of both moderate‐ and high‐autistic‐trait groups were negative for the intercept, which showed lower adaptive scores at the first measurement (age 2.7 years) compared with the low‐autistic‐trait group (Table 3). The main effects of female sex were positive for intercept and slope, and negative for the quadratic term in communication compared with the male sex (Table 3). The group‐by‐sex interactions were not significant in the other two domains. Detailed results of LGCM on adaptive behaviors are shown in Table 3, and estimated and observed trajectories are shown in Figs. S7 and S8, in Appendix S1, respectively.

**Table 3:** Estimated standardized coefficients in each domain of adaptive behaviors using the latent growth curve modeling

*β* (95% CI)			
	Communication	Daily living skills	Socialization
Female sex by moderate‐autistic‐trait group interactions			
Intercept	−0.001 (−0.11, 0.12)	0.001 (−0.10, 0.11)	0.04 (−0.07, 0.14)
Slope	−0.05 (−0.18, 0.09)	−0.08 (−0.23, 0.05)	−0.003 (−0.16, 0.16)
Quadratic	0.05 (−0.09, 0.19)	0.06 (−0.09, 0.23)	0.02 (−0.15, 0.19)
Female sex by high‐autistic‐trait group interactions			
Intercept	0.14 (0.02, 0.26)*	0.07 (−0.04, 0.19)	0.01 (−0.11, 0.13)
Slope	−0.25 (−0.40, −0.11)*	−0.03 (−0.19, 0.14)	0.03 (−0.12, 0.18)
Quadratic	0.21 (0.07, 0.37)*	−0.06 (−0.23, 0.13)	−0.03 (−0.20, 0.13)
Main effects of moderate‐autistic‐trait group (low is reference)			
Intercept	−0.13 (−0.24, −0.02)*	−0.13 (−0.24, −0.02)*	−0.24 (−0.35, −0.12)*
Slope	−0.12 (−0.26, 0.01)	−0.06 (−0.21, 0.08)	−0.14 (−0.29, 0.001)
Quadratic	0.07 (−0.06, 0.21)	0.04 (−0.11, 0.20)	0.03 (−0.12, 0.19)
Main effects of high‐autistic‐trait group (low is reference)			
Intercept	−0.22 (−0.34, −0.10)*	−0.19 (−0.30, −0.08)*	−0.15 (−0.27, −0.04)*
Slope	0.09 (−0.04, 0.24)	0.01 (−0.15, 0.17)	−0.15 (−0.34, −0.004)*
Quadratic	−0.12 (−0.27, 0.02)	0.002 (−0.17, 0.19)	0.05 (−0.11, 0.24)
Main effects of female sex (male is reference)			
Intercept	0.26 (0.17, 0.35)*	0.20 (0.11, 0.28)*	0.21 (0.12, 0.30)*
Slope	0.24 (0.13, 0.36)*	0.08 (−0.04, 0.20)	0.08 (−0.04, 0.19)
Quadratic	−0.24 (−0.36, −0.11)*	−0.07 (−0.19, 0.07)	−0.06 (−0.18, 0.05)

Abbreviations: β, standardized coefficient; 95% CI, 95% confidence interval.

* *p* < 0.05.

### Expressive language and communications in females and males with high autistic traits

**Fig. 3:**
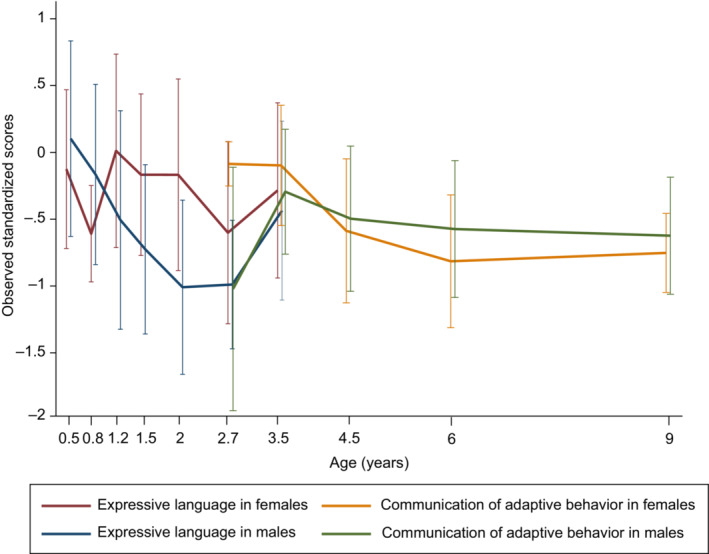
Observed trajectories of expressive language and communication of adaptive behavior in females and males in the high‐autistic‐trait group.

Figure 3 shows the observed trajectories of expressive language and communication, in which significant group‐by‐sex interactions were seen for females and males in the high‐autistic‐trait group. Expressive language represents a cognitive function that is directly assessed by the evaluator, while communication represents language skills used in everyday situation rated by parents. The figure shows that between the ages of 2.7 and 3 years, when the assessment timings overlapped, the directly assessed language function and adaptive use of language (communication) assessed by parent were consistent for males, but there was a discrepancy between the two for females in this group.

### Attrition

Missing values in motor, cognition, and adaptive behaviors were dealt with multiple imputation methods. To examine whether the results were replicated using a different method of handling missing values, we also used the full information maximum likelihood (FIML) approach. The results of the two methods were almost identical.

## Discussion

The present study is the first longitudinal study to examine sex differences in developmental trajectories for different groups with varying levels of autistic traits in the general population, regardless of clinical diagnosis. The results revealed that females with high autistic traits had neurodevelopmental trajectories different from their male counterparts. The difference manifested in expressive language measured by the MSEL at around 2 years of age and in the communication domain of adaptive behavior measured by the VABS‐II after 3 years of age. Males with high autistic traits showed a temporary decline in expressive language around the age of 2 years, but females with high autistic trait showed no apparent decline compared to their male counterparts. In the communication domain of adaptive behavior, males with high autistic traits showed a slight improvement around the age of 3 years, but then showed a gradual decline. Meanwhile, females with high autistic traits showed a notable decline in communication after the age of 3.5 years. Before age 3, in females with high autistic traits, there tended to be a discrepancy between expressive language as assessed directly by the evaluator and communication (adaptive use of language) as assessed by the parents.

These results suggest that language assessment may be of interest as an early marker of ASD in males, while the assessment of adaptive behaviors, especially communication (adaptive use of language) in everyday situations, and examining the discrepancy between cognitive functions and adaptive behaviors may be worth consideration for early detection of ASD in females. Future research examining if these patterns are observed in children formally diagnosed with ASD is of interest. If the findings of this study are replicated, early intervention for communication skills required in daily living situations could be developed for females with high autistic traits even if they show no apparent decline in the expressive language function.

Recent studies examining early motor and cognitive functions have not reported sex differences in children diagnosed with ASD,^31^ which is inconsistent with our results. This may be due to differences in the population comprising the sample; previous studies have included children diagnosed with ASD, while the present study included children who would not have an ASD diagnosis despite having higher autistic traits than their same‐sex peers. In the present study, females with high autistic traits did not show an apparent decline in early expressive language unlike their male counterparts, who showed a temporary decline at around 2 years of age and a slight improvement around 3 years of age. Previous studies also found that females with high autistic traits demonstrated no obvious difficulties in communication before the age of 3 years, but they also reported that the difficulties become apparent with age. For example, Mahendiran *et al*.^32^ reported that adaptive functions were lower in females compared to males with ASD at 10–12 years of age, despite females performing better at younger ages (8–10 years). Wood‐Downie *et al*.^33^ also reported in their meta‐analysis that the difference between females with and without ASD became more evident with age. These findings suggest that even if females have no difficulty in acquiring language functions, they have difficulties in using them in communicative settings, and their undiagnosed condition may contribute to increased difficulties.

Our findings for males with high autistic traits are consistent with previous studies. For example, a recent large national cohort of individuals with autism found that parents of males reported consistent speech and language concerns, indicating speech as their first concern.^34^ Although sex differences were not examined, Pickles *et al*.^35^ reported that there was a catch‐up trajectory in language function in children with ASD by the age of 3, which was similar to our results. Concurrent with the slight improvement in expressive language around 3 years of age, males with high autistic traits showed a similar trend in the communication domain of adaptive behaviors. However, this temporary improvement was followed by a gradual decline during school age.

In the present study, various aspects of early development were examined longitudinally, including females who might be overlooked by the current diagnostic criteria of ASD. However, several limitations should be considered. First, the groupings in the present study were based solely on the autistic traits measured at the age of 9 years. Autism is sometimes conceptualized as an innate or inborn condition and the presence of genetic and other factors during the earliest years of life is considered to have a significant contribution to its onset. However, autistic social traits develop during childhood and adolescence, and their trajectories differ between females and males.^36^, ^37^ We focused on autistic traits at the age of 9 years considering the average age of ASD diagnosis, but future studies should take into account sex differences in the manifestation of autistic traits at different age ranges within the development. Second, although autistic traits are continuously distributed and have no natural cut‐point between autism and subclinical social difficulties, we have used grouping based on the statistical cut‐points. Additionally, grouping reduced sample size of the high‐autistic‐trait group, which might have compromised the stability of the estimates. To address this limitation, we estimated the models including the SRS–2 raw scores as continuous variables, rather than dividing them into groups. The results showed that the interaction of sex and autistic traits was significant only in the communication domain of adaptive behavior. The interaction of sex and autistic traits in expressive language was not significant. Furthermore, we estimated the models including autistic‐trait‐groups categorized by the same cut‐point for females and males, rather than sex‐specific cut‐points. Here again, the results showed that the interaction of sex and clinical‐autistic‐trait group (raw scores above +2.5 SD) was significant only in the communication domain of adaptive behavior, although the intercept was not significant (Tables S4 and S5, in Appendix S1). The inconsistent findings in expressive language suggest that females with high autistic trait have similar difficulties in expressive language as their male counterparts. The consistent findings in the communication domain suggest that in the general population, females with quantitatively higher autistic traits may have a different developmental trajectory of communication than males with similar traits. In any case, results derived from a small sample are susceptible to between‐subject variability inherent to the developmental milestone measures, and it is possible that the sex differences are a function of the severity of autistic traits rather than sex; thus, the results need to be confirmed in a larger sample for clinical application. Third, some measures that were reported by parents might be biased, except for motor and cognitive functions, which were directly evaluated by trained evaluators. However, the autistic traits seen in daily life and adaptive functioning are better perceived by the parents living with the child than by outside evaluators. Kamio *et al*.^38^ found that parental ratings of autistic traits were higher than teacher ratings for females with ASD, and it was suggested that autistic traits can be underestimated in school settings, especially for females with mild autistic traits but without IDs. Fourth, information on clinical diagnosis of ASD was not available for the entire sample at age 9, and parent‐reported SRS–2 scores cannot be used for diagnosis. Even if information on clinical diagnosis could be obtained, given the currently reported prevalence and sex differences, it is expected that only a few females in this sample would be diagnosed with ASD. Finally, 38.4% of the participants were excluded from the analyses in this study because they were not assessed at 9 years of age. When comparing the demographic characteristics between the groups included and excluded from the analyses, some variables differed between these groups (Table S6, in Appendix S1). Children with younger parents and mothers with lower levels of education were excluded from the analyses, which may have affected the results of this study. Considering these limitations, the results of this study need to be confirmed in a larger sample, but results obtained from the general population should be discussed and compared with those obtained from the clinical population.

## Conclusions

The present study identified that females and males with high autistic traits (approximately 3% of the distribution for each sex) at the age of 9 years showed different developmental trajectories. Differences appeared in expressive language at around 2 years of age and in adaptive communication after 3 years of age. The results suggest that early intervention for adaptive communication would be beneficial for females with autistic traits even if they show no apparent decline in language functions. Meanwhile, males with high autistic traits may show early signs, especially in expressive language, at around 2 years of age. The current results suggest the effectiveness of screening at the appropriate time, as well as the formation of early support system, tailored for possible sex differences in developmental trajectories.

## Funding Information

This work was supported by AMED (JP21gk0110039) and the Japan Society for the Promotion of Science, Grants‐in‐Aid for Scientific Research (20K07941, 19H03582, 21KK0145, 22H00492). The funders had no role in the study design, data collection and analysis, decision to publish, or preparation of the manuscript.

## Disclosure statement

The authors declare no conflict of interest.

## Author contributions

T.N. and K.J.T. were responsible for study conceptualization and design and formulated the specific research question. T.N. conducted the statistical analyses and drafted the initial manuscript. N.T., Y.N., N.T., A.S., and K.J.T. supervised and critically revised the manuscript. A.O., T.H., T.I., C.N., S.R., S.U., and M.W. contributed to the interpretation of the results. T.N., A.O., T.H., T.I., C.N., and K.J.T. conducted the data acquisition. All authors contributed to editing the manuscript and approved the final version.

## Supporting information


**Appendix S1.** Supporting information

## Data Availability

The data that support the findings of this study are available on request from the corresponding author, K.J.T. The data are not publicly available as they contain information that could compromise the privacy of research participants.
