# Supplementary material for: Sex differences in neurodevelopmental trajectories in children with different levels of autistic traits
Source: Psychiatry Clin Neurosci. 2023 Feb 6;77(5):282–9. doi: 10.1111/pcn.13529 (PMC11488628; doi:10.1111/pcn.13529)
Supplement: Supplementary file 1 — Appendix S1. Supporting information [file PCN-77-282-s001.docx]

Table S1. Comparison of demographic characteristics between participants in HBC Study and national statistics of Japan

|  | HBC Study participants | National statistics of Japan^1^ |
| --- | --- | --- |
| Child characteristics (n = 1258) |  |  |
| Male sex; n (%) | 648 (52%) | 527,657 (51%) |
| Birthweight (g); mean (SD) | 2942 (435) | 3000 |
| Low birthweight; n (%) | 160 (12.7%) | 98,624 (10%) |
| Gestational age (week); mean (SD) | 38.9 (1.6) | Data not available |
| Preterm birth; n (%) | 82 (6.5%) | 59,235 (5.8%) |
| Parity (primipara); n (%) | 626 (50%) | 481,418 (47%) |
| Parity (multipara); n (%) | 632 (50%) | 547,858 (53%) |
| Twins; n (%) | 38 (3%) | 19,901 (2%) |
| SRS-2 total raw score; mean (SD) | 34.3 (18.1) | 32.5 (18.2)^2^ |
| SRS-2 total T-score; mean (SD) | 50.9 (9.9) | 50.0 (10.0) |
| WISC-Ⅳ full scale IQ; mean (SD) | 101.6 (14.0) | 100.0 (15.0) |
| Parental characteristics (n = 1138) |  |  |
| Mother's age at birth (y); mean (SD) | 31.4 (5.1) | 31.2 |
| Father's age at birth (y); mean (SD) | 33.3 (5.9) | 33.2 |
| Mother's educational year (y); mean (SD) | 13.8 (2.0) | Data not available |
| Father's educational year (y); mean (SD) | 14.1 (2.7) | Data not available |
| Annual household income (million JPY); mean (SD) | 6.09 (2.82) | 6.28 |

Abbreviations: HBC Study, Hamamatsu Birth Cohort Study for Mothers and Children; SD, standard deviation; SRS-2, Social Responsiveness Scale, Second Edition; WISC-Ⅳ, Wechsler Intelligence Scale for Children-Fourth Edition

Reference

1 Ministry of Health Labour and Welfare. Vital Statistics in Japan. 2018. <https://www.mhlw.go.jp/english/database/db-hw/dl/81-1a2en.pdf> (9 August 2021, date last accessed)

2 Kamio, Y., Inada, N., Moriwaki, A., Kuroda, M., Koyama, T., Tsujii, H., Kawakubo, Y., Kuwabara, H., Tsuchiya, K. J., Uno, Y. & Constantino, J. N. (2013). Quantitative autistic traits ascertained in a national survey of 22 529 Japanese schoolchildren. *Acta Psychiatr Scand*, 128, 45-53.


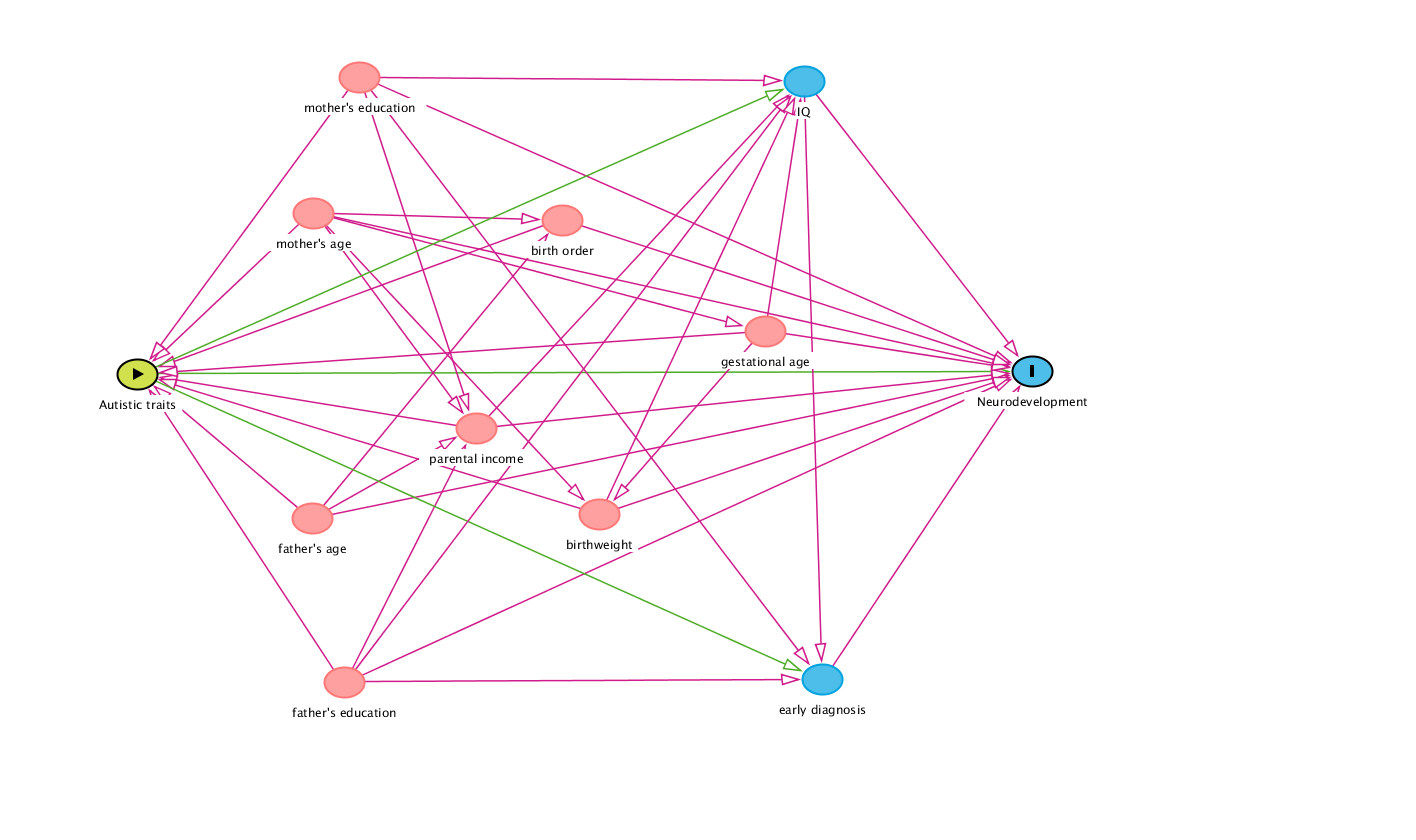
Figure S1. Directed acyclic graph for autistic traits and neurodevelopment

In this model, autistic traits are assumed to be inborn condition based on the perspective that genetic and other effects occurring during the earliest times of life play a significant contributory role to the presentation of autism. The green circle is the exposure (autistic traits). The blue circles are either ancestors of the outcome or the outcome (neurodevelopment). The red circles are both ancestors of the exposure and the outcome. The green lines are causal pathways, and pink lines are biasing pathways.


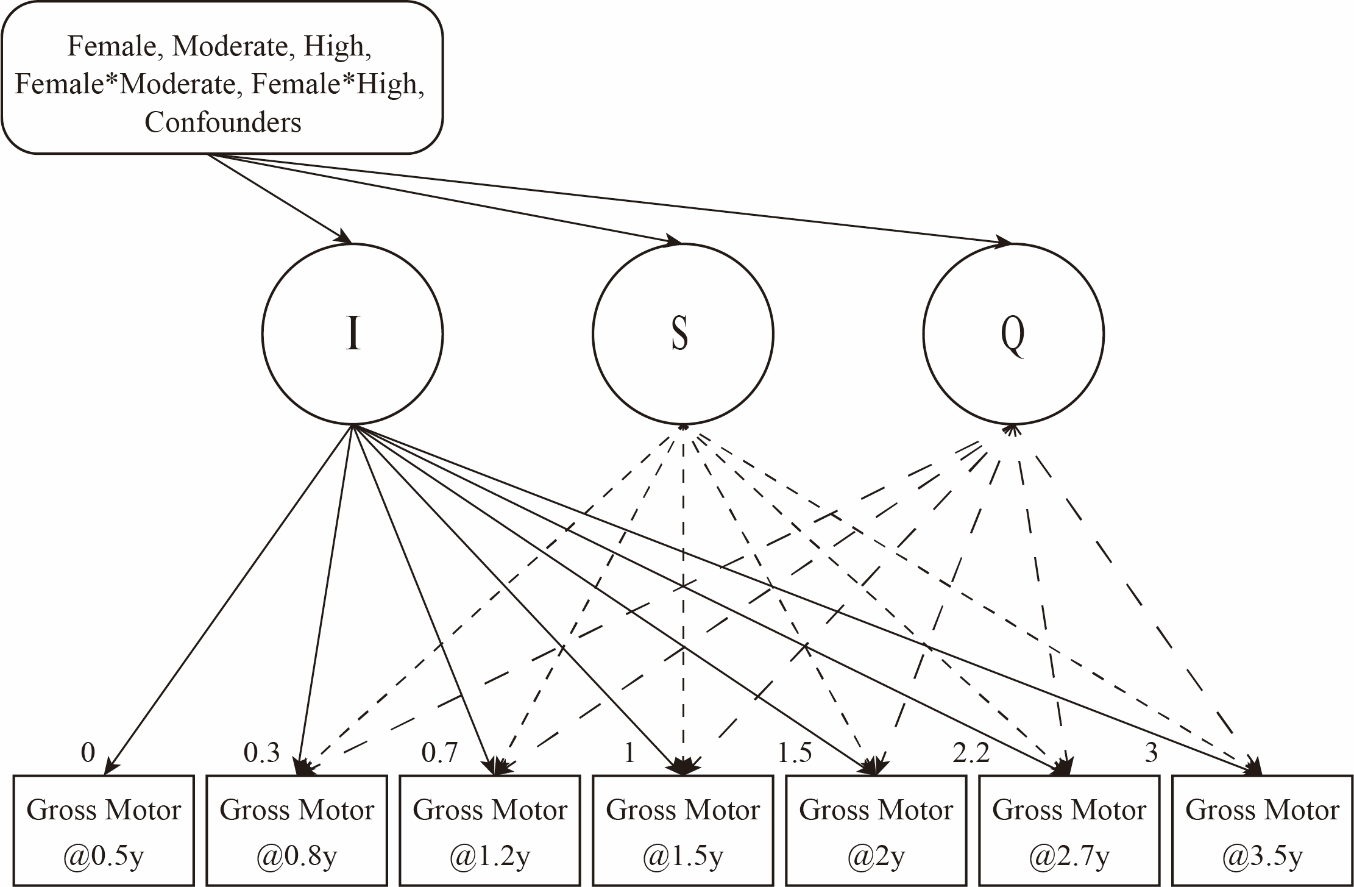
Figure S2. Latent growth curve model for gross motor


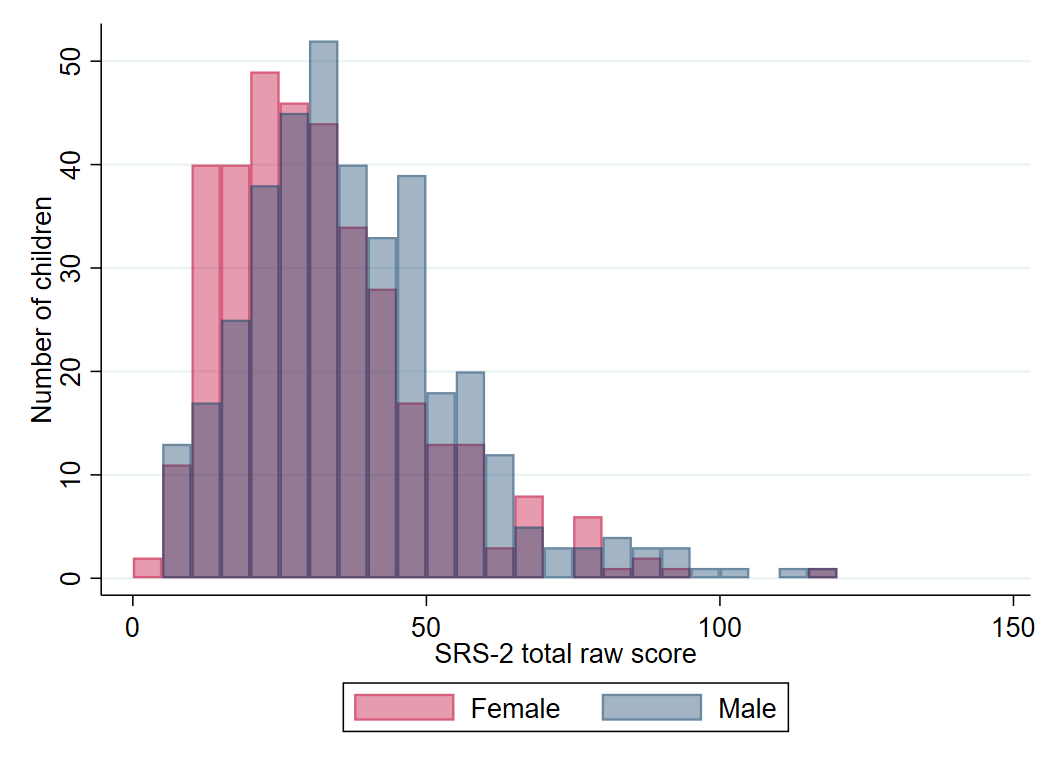


Figure S3. Distribution of Social Responsiveness Scale, Second Edition (SRS-2) total raw scores rated by caregivers participated in HBC Study


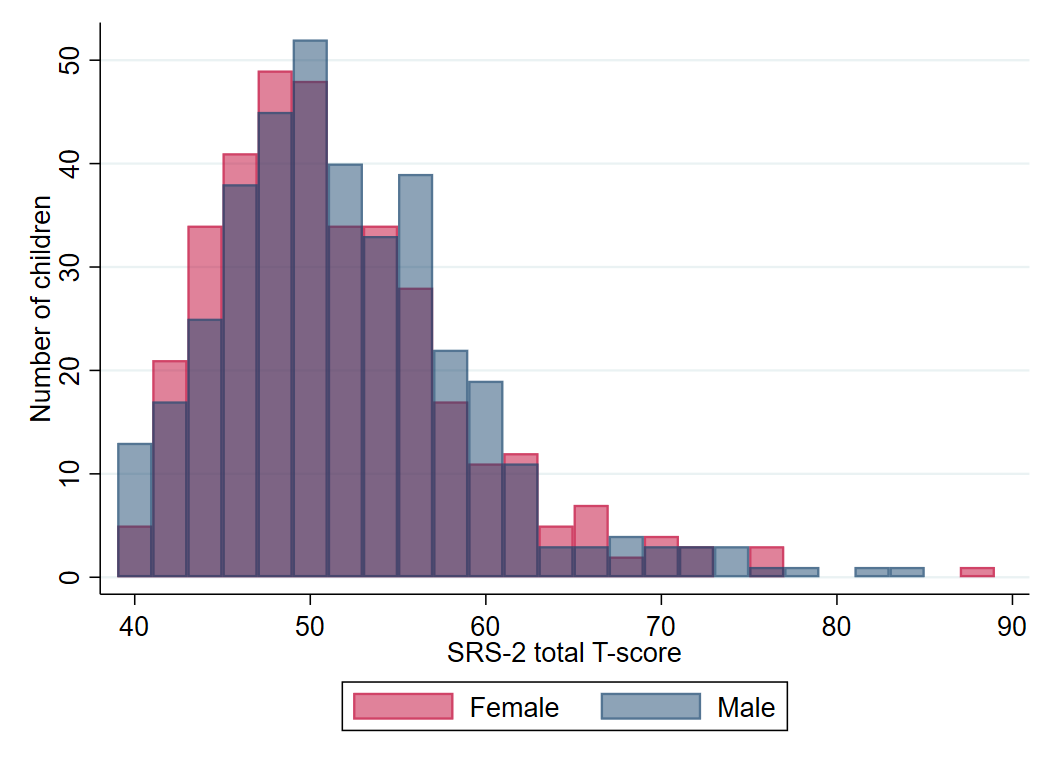


Figure S4. Distribution of Social Responsiveness Scale, Second Edition (SRS-2) total T-scores rated by caregivers participated in HBC Study

Table S2. Sex differences in each autistic-trait group in demographic characteristics

|  | Low-autistic-trait group | | | Moderate-autistic-trait group | | | High-autistic-trait group | | |  |
| --- | --- | --- | --- | --- | --- | --- | --- | --- | --- | --- |
| Autistic trait group | Female (n=345, 85.6%) | Male (n=347, 82.4%) | Difference | Female (n=46, 11.4%) | Male (n=63, 15.0%) | Difference | Female (n=12, 3.0%) | Male (n=11, 2.6%) | Difference | |
| SRS-2 total raw score; mean (SD) | 26.0 (10.7) | 30.9 (11.5) | β=-.22, p<.001 | 57.4 (7.0) | 63.0 (8.3) | β=-.34, p<.001 | 84.0 (12.3) | 95.8 (11.3) | β=-.46, p=.03 | |
| SRS-2 total T-score; mean (SD) | 47.0 (6.2) | 48.3 (6.0) | β=-.11, p=.005 | 65.4 (4.0) | 65.1 (4.3) | ns | 81.0 (7.0) | 82.4 (5.8) | ns | |
| WISC-Ⅳ full scale IQ; mean (SD) | 104.1 (12.7) | 101.0 (13.9) | β=.12, p=.002 | 98.5 (16.0) | 98.3 (15.8) | ns | 91.2 (11.2) | 91.7 (16.4) | ns | |
| Background characteristics of children | | | | | | | | | | |
| Birthweight; mean (SD) | 2872.5 (439.3) | 2966.8 (471.3) | β=-.10, p=.007 | 2932.1 (444.0) | 2972.7 (503.7) | ns | 2897.5 (370.5) | 3031.5 (505.8) | ns | |
| Gestational age; mean (SD) | 38.9 (1.6) | 38.8 (1.7) | ns | 39.1 (1.5) | 39.0 (1.7) | ns | 38.8 (1.4) | 39.0 (1.1) | ns | |
| Parity (primiparity); n (%) | 173 (50.1) | 160 (46.1) | ns | 28 (60.9) | 37 (58.7) | ns | 8 (66.7) | 7 (63.6) | ns | |
| Background characteristics of parents | | | | | | | | | | |
| Mother's age at birth; mean (SD) | 31.8 (4.9) | 31.9 (5.0) | ns | 33.1 (5.4) | 32.4 (5.6) | ns | 31.4 (6.2) | 31.3 (4.0) | ns | |
| Father's age at birth; mean (SD) | 33.7 (5.7) | 33.2 (5.6) | ns | 35.3 (5.9) | 34.7 (6.8) | ns | 36.9 (5.2) | 32.9 (5.6) | ns | |
| Mother's educational history; mean (SD) | 14.1 (1.9) | 14.0 (1.9) | ns | 13.7 (1.9) | 13.8 (1.5) | ns | 13.3 (1.7) | 13.8 (1.3) | ns | |
| Father's educational history; mean (SD) | 14.3 (2.6) | 14.1 (2.5) | ns | 14.0 (2.7) | 14.0 (3.1) | ns | 12.7 (2.7) | 14.3 (2.0) | ns | |
| Annual household income at birth | 6.5 (0.3) | 5.9 (0.3) | β=.11, p=.005 | 6.3 (0.3) | 5.9 (0.3) | ns | 5.6 (0.2) | 6.0 (0.2) | ns | |

SD, standard deviation; SRS-2, Social Responsiveness Scale, Second Edition; WISC-Ⅳ, Wechsler Intelligence Scale for Children-Fourth Edition

There were no missing values for all variables.

Table S3. Group differences in demographic characteristics

|  | Stats | | | Group differences | | |  |
| --- | --- | --- | --- | --- | --- | --- | --- |
| Autistic trait group | Low (n=692, 84.0%) | Moderate (n=109, 13.2%) | High (n=23, 2.8%) | Low vs. Moderate | Low vs. High | Moderate vs. High |  |
| SRS-2 total raw score; mean (SD) | 28.5 (11.4) | 60.6 (8.2) | 89.7 (13.0) | β=.60, p<.001 | β=.56, p<.001 | β=.27, p<.001 |  |
| SRS-2 total T-score; mean (SD) | 47.7 (6.1) | 65.3 (4.2) | 81.7 (6.4) | β=.60, p<.001 | β=.57, p<.001 | β=.27, p<.001 |  |
| WISC-Ⅳ full scale IQ; mean (SD) | 102.6 (13.4) | 98.4 (15.8) | 91.4 (13.6) | β=-.10, p=.003 | β=-.13, p<.001 | β=-.08, p=.03 |  |
| Background characteristics of children | | | | | | | |
| Birthweight; mean (SD) | 2919.8 (457.7) | 2955.6 (477.7) | 2961.6 (435.4) | ns | ns | ns |  |
| Gestational age; mean (SD) | 38.9 (1.7) | 39.1 (1.6) | 38.9 (1.3) | ns | ns | ns |  |
| Parity (primiparity), n (%) | 333 (48.1) | 65 (59.6) | 15 (65.2) | OR=1.6, p=.03 | ns | ns |  |
| Background characteristics of parents | | | | | | | |
| Mother's age at birth; mean (SD) | 31.9 (4.9) | 32.7 (5.5) | 31.3 (5.1) | ns | ns | ns |  |
| Father's age at birth; mean (SD) | 33.5 (5.7) | 34.9 (6.4) | 35.0 (5.7) | β=.09, p=.01 | ns | ns |  |
| Mother's educational history; mean (SD) | 14.0 (1.9) | 13.8 (1.7) | 13.5 (1.5) | ns | ns | ns |  |
| Father's educational history; mean (SD) | 14.2 (2.9) | 14.0 (2.9) | 13.4 (2.5) | ns | ns | ns |  |
| Annual household income at birth; mean (SD) | 619.7 (286.6) | 608.9 (297.1) | 577.0 (180.3) | ns | ns | ns |  |

SD, standard deviation; SRS-2, Social Responsiveness Scale, Second Edition; WISC-Ⅳ, Wechsler Intelligence Scale for Children-Fourth Edition

There were no missing values for all variables.


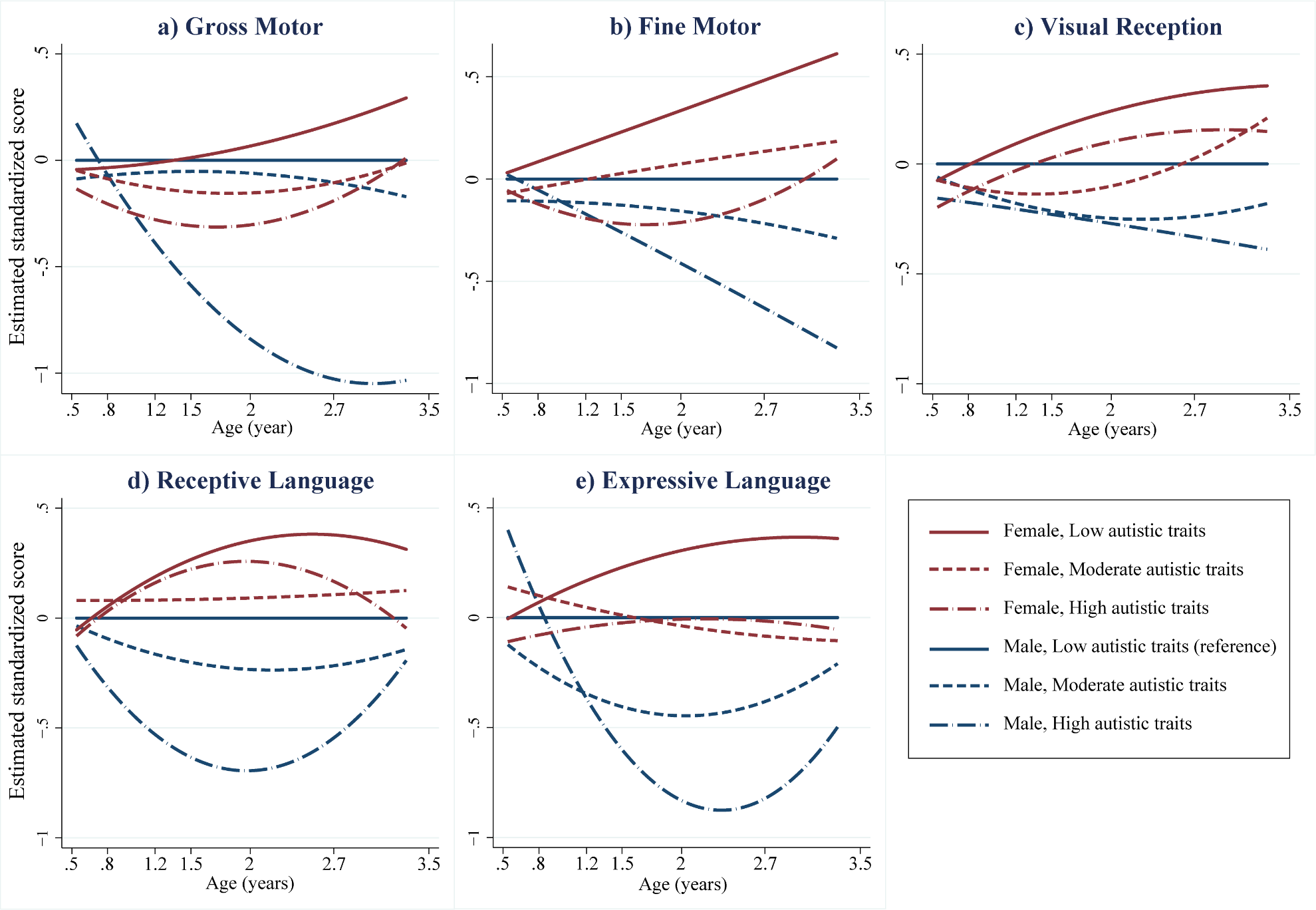
Figure S5. Estimated standardised scores in motor and cognitive functions: a) gross motor, b) fine motor, c) visual reception, d) receptive language, and e) expressive language.


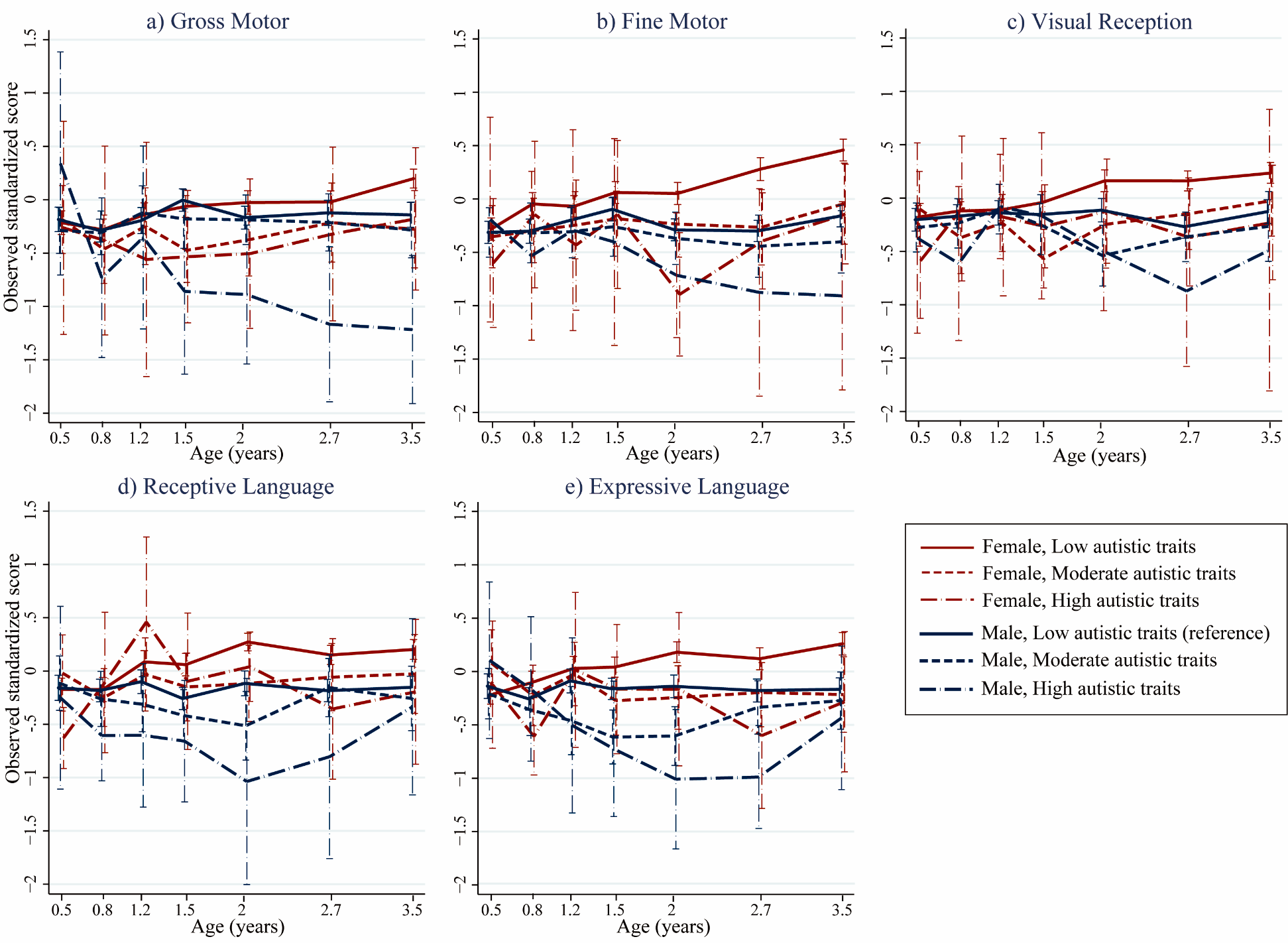
Figure S6. Observed standardised scores in motor and cognitive functions: a) gross motor, b) fine motor, c) visual reception, d) receptive language, and e) expressive language.


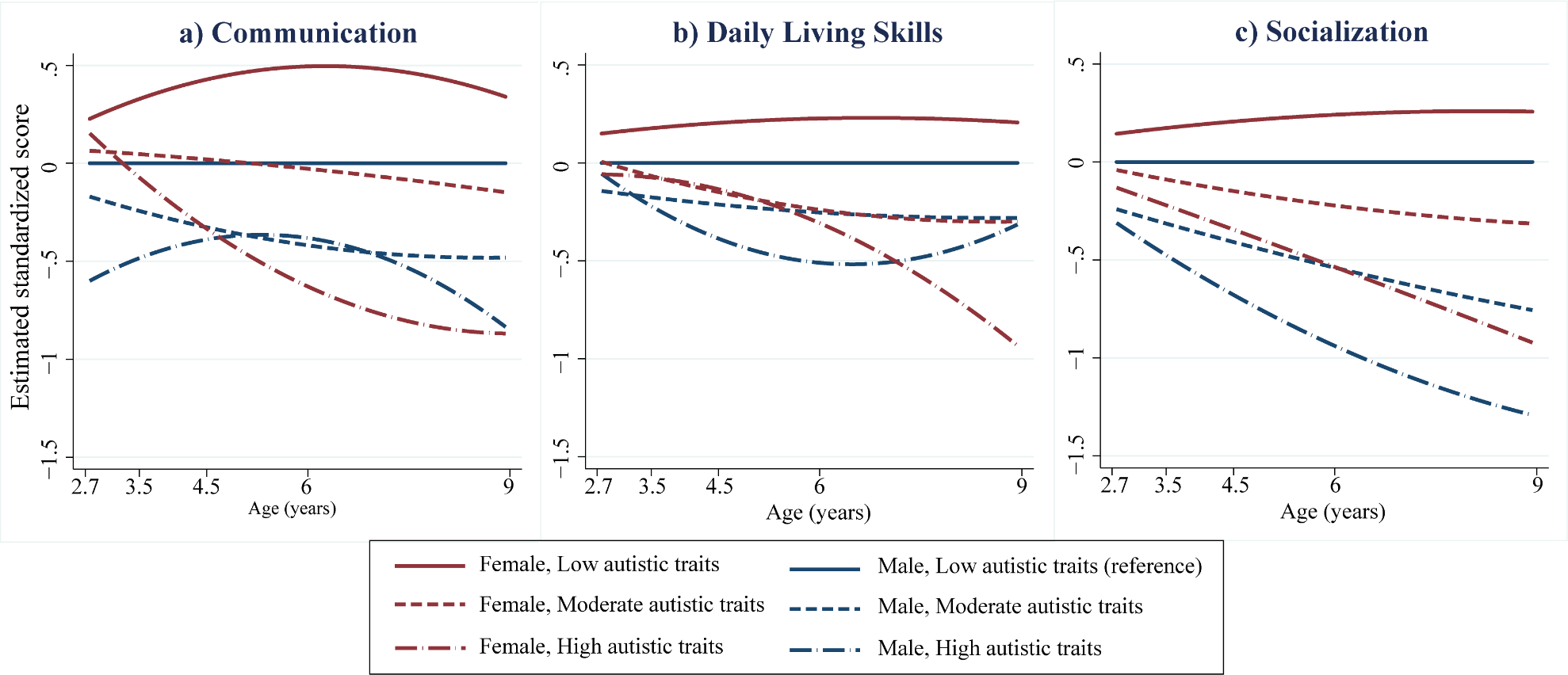


Figure S7. Estimated standardised scores in each domain of adaptive behaviours: a) communication, b) daily living skills, and c) socialisation.


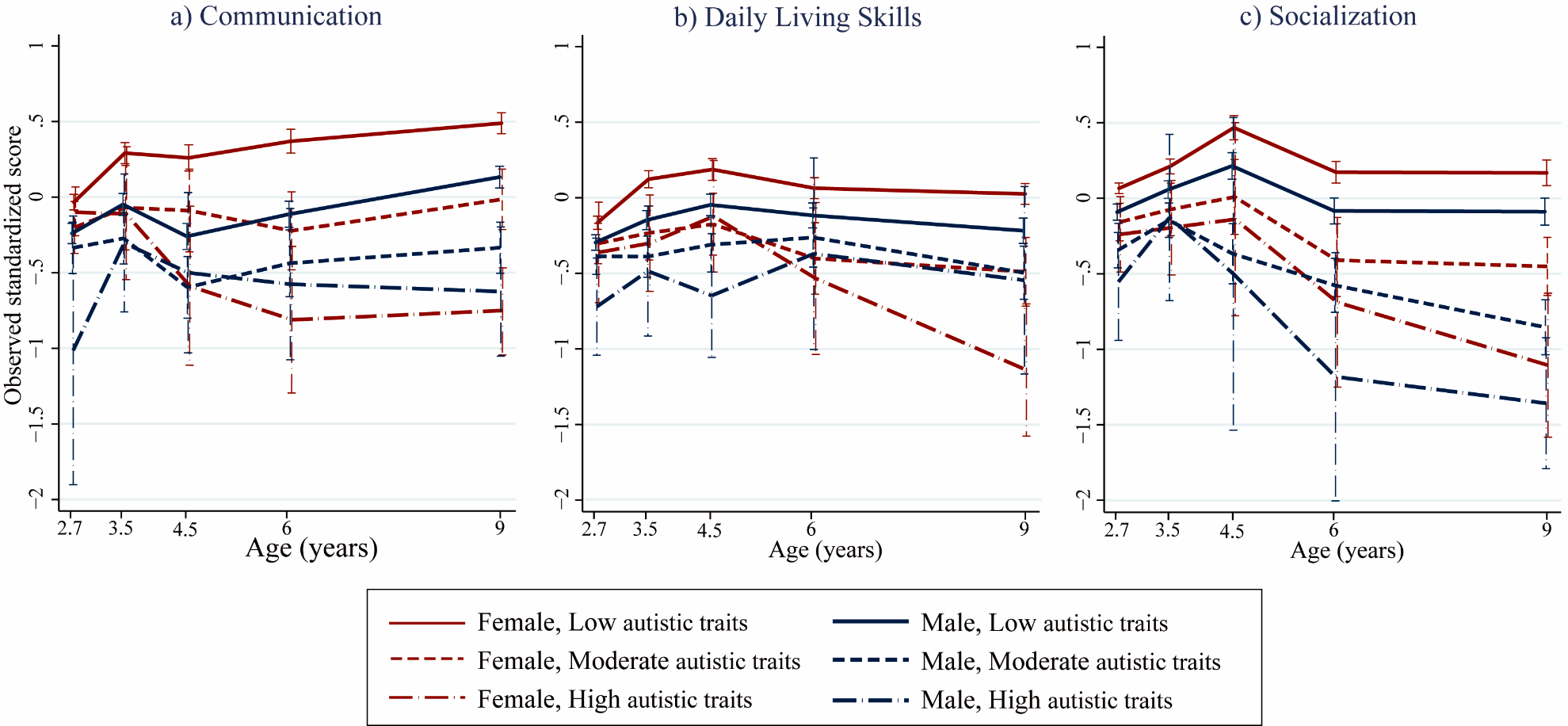


Figure S8. Observed standardised scores in each domain of adaptive behaviours: a) communication, b) daily living skills, and c) socialisation.

Table S4. Estimated Values in Each Domain of MSEL Using the Same SRS-2 Cut-point for Females and Males

| β (95% CI) | | | | | |
| --- | --- | --- | --- | --- | --- |
|  | Gross motor | Fine motor | Visual reception | Receptive language | Expressive language |
| Female sex by subclinical-autistic-trait group interactions | | | | | |
| Intercept | .10 (-.30, .50) | .12 (-.23, .43) | .27 (-.08, .63) | .29 (-.09, .63) | .23 (-.15, .61) |
| Slope | -.33 (-.95, .25) | -.42 (-1.00, .20) | -.38 (-.95, .14) | -.30 (-.88, .23) | .02 (-.54, .59) |
| Quadratic | .09 (-.11, .28) | .11 (-.08, .30) | .10 (-.07, .26) | .07 (-.09, .24) | -.06 (-.23, .10) |
| Female sex by clinical-autistic-trait group interactions | | | | | |
| Intercept | -.86 (-1.71, .06) | -.28 (-1.16, .49) | -.08 (-.98, .75) | -.01 (-.83, .85) | -.65 (-1.53, .38) |
| Slope | 1.23 (-.41, 2.71) | -.20 (-1.64, 1.35) | .13 (-1.40, 1.43) | .66 (-.65, 1.88) | 1.15 (-.31, 2.65) |
| Quadratic | -.24 (-.69, .25) | .12 (-.34, .55) | -.01 (-.42, .46) | -.33 (-.70, .08) | -.34 (-.80, .06) |
| Main effects of subclinical-autistic-trait group (typical is reference) | | | | | |
| Intercept | -.09 (-.35, .15) | -.12 (-.32, .11) | -.03 (-.27, .21) | -.03 (-.27, .21) | -.13 (-.39, .09) |
| Slope | .08 (-.32, .46) | .02 (-.34, .36) | -.26 (-.64, .10) | -.24 (-.60, .12) | -.38 (-.77, .04) |
| Quadratic | -.04 (-.16, .08) | -.02 (-.13, .09) | .08 (-.04, .20) | .07 (-.03, .18) | .13 (.01, .24) |
| Main effects of clinical-autistic-trait group (typical is reference) | | | | | |
| Intercept | .12 (-.42, .58) | .07 (-.36, .56) | -.19 (-.61, .31) | -.17 (-.64, .31) | .32 (-.25, .83) |
| Slope | -.83 (-1.56, -.05)^*^ | -.35 (-1.11, .41) | -.11 (-.87, .66) | -.68 (-1.41, .06) | -1.41 (-2.25, -.63)^*^ |
| Quadratic | .17 (-.07, .41) | -.003 (-.24, .24) | -.01 (-.26, .23) | .22 (-.004, .44) | .40 (.18, .64)^*^ |
| Main effects of female sex (male is reference) | | | | | |
| Intercept | -.04 (-.16, .10) | .02 (-.12, .14) | -.09 (-.22, .05) | -.06 (-.20, .07) | .004 (-.14, .14) |
| Slope | .02 (-.19, .20) | .23 (.03, .45)^*^ | .31 (.08, .55)^*^ | .44 (.24, .65)^*^ | .29 (.06, .52)^*^ |
| Quadratic | .04 (-.02, .10) | -.01 (-.08, .05) | -.05 (-.13, .01) | -.11 (-.18, -.06)^*^ | -.06 (-.13, .01) |

*Note*. *p<.05; MSEL, Mullen Scales of Early Learning; β, standardized coefficient; 95% CI, 95% confidence interval

Autistic trait groups; Typical: SRS-2 raw scores below +1 standard deviation (SD) (female, n=358 (88.8%); male, n=347 (82.4%)), Subclinical: from +1 to +2.5 SD (female, n=40 (9.9%); male, n=60 (14.3%)), Clinical: above +2.5 SD (female, n=5 (1.2%); male, n=14 (3.3%))

Table S5. Estimated Values in Each Domain of Adaptive Behaviors Using the Same SRS-2 Cut-point for Females and Males

|  | β (95% CI) | | |
| --- | --- | --- | --- |
|  | Communication | Daily Living Skills | Socialization |
| Female sex by subclinical-autistic-trait group interactions | | | |
| Intercept | .07 (-.15, .32) | -.12 (-.30, .06) | -.04 (-.22, .14) |
| Slope | -.12 (-.31, .06) | -.02 (-.18, .16) | .04 (-.15, .23) |
| Quadratic | .01 (-.02, .04) | .001 (-.03, .03) | .004 (-.04, .03) |
| Female sex by clinical-autistic-trait group interactions | | | |
| Intercept | .35 (-.22, .90) | .39 (-.09, .84) | .11 (-.33, .51) |
| Slope | -.02 (-.03, -.01)^*^ | -.09 (-.53, .34) | -.04 (-.50, .41) |
| Quadratic | .09 (.01, .16)^*^ | -.03 (-.09, .04) | .004 (-.07, .08) |
| Main effects of subclinical-autistic-trait group (typical is reference) | | | |
| Intercept | -.18 (-.33, -.03)^*^ | -.12 (-.24, .001) | -.24 (-.36, -.13)^*^ |
| Slope | -.11 (-.24, .01) | -.05 (-.17, .07) | -.09 (-.24, .01) |
| Quadratic | .01 (-.01, .03) | .004 (-.01, .02) | .002 (-.01, .03) |
| Main effects of clinical-autistic-trait group (typical is reference) | | | |
| Intercept | -.47 (-.76, -.18)^*^ | -.48 (-.70, -.24)^*^ | -.30 (-.53, -.09)^*^ |
| Slope | .15 (-.09, .40) | -.001 (-.22, .23) | -.24 (-.47, -.03)^*^ |
| Quadratic | -.03 (-.07, .002) | .001 (-.04, .04) | .01 (-.02, .05) |
| Main effects of female sex (male is reference) | | | |
| Intercept | .22 (.14, .30)^*^ | .15 (.09, .22)^*^ | .15 (.08, .21)^*^ |
| Slope | .15 (.07, .22)^*^ | .03 (-.03, .09) | .04 (-.03, .10) |
| Quadratic | -.02 (-.03, .01)^*^ | -.004 (-.01, .01) | -.004 (-.01, .01) |

*Note*. *p<.05; MSEL, Mullen Scales of Early Learning; β, standardized coefficient; 95% CI, 95% confidence interval

Autistic trait groups; Typical: SRS-2 raw scores below +1 standard deviation (SD) (female, n=358 (88.8%); male, n=347 (82.4%)), Subclinical: from +1 to +2.5 SD (female, n=40 (9.9%); male, n=60 (14.3%)), Clinical: above +2.5 SD (female, n=5 (1.2%); male, n=14 (3.3%))

Table S6. Comparison of the demographic characteristics between the groups included and excluded from the analyses

|  | Included in analyses (n=824) | Excluded from analyses (n=434) | Difference testing | Effect size |
| --- | --- | --- | --- | --- |
| Child characteristics |  |  |  |  |
| Male sex; n (%) | 421 (51.1%) | 226 (52.1%) | χ^2^ = .11, p = .74 |  |
| Birthweight (g); mean (SD) | 2925.7 (459.4) | 2968.5 (395.6) | t = -1.65, p = .10 |  |
| Gestational age (week); mean (SD) | 38.9 (1.65) | 39.0 (1.51) | t = -1.09, p = .27 |  |
| Parity (primipara); n (%) | 413 (50.1%) | 213 (49.1%) | χ^2^ = .12, p = .73 |  |
| Parental characteristics (n = 1138) |  |  |  |  |
| Mother’s age at birth (y); mean (SD) | 32.0 (5.0) | 30.5 (5.0) | t = 4.92, p < .001 | *d* = .29 |
| Father’s age at birth (y); mean (SD) | 33.7 (5.8) | 32.4 (5.8) | t = 3.89, p < .001 | *d* = .23 |
| Mother’s educational year (y); mean (SD) | 14.0 (1.9) | 13.5 (2.2) | t = 3.99, p < .001 | *d* = .24 |
| Father’s educational year (y); mean (SD) | 14.2 (2.6) | 14.0 (2.8) | t = 1.00, p = .32 |  |
| Annual household income (million JPY); mean (SD) | 6.17 (2.85) | 5.78 (2.78) | t = 2.35, p = .02 | *d* = .24 |

SD, standard deviation
